# Stratified split sampling of electronic health records

**DOI:** 10.1186/s12874-023-01938-0

**Published:** 2023-05-25

**Authors:** Tianyao Huo, Deborah H. Glueck, Elizabeth A. Shenkman, Keith E. Muller

**Affiliations:** 1 Department of Health Outcomes and Biomedical Informatics, College of Medicine, University of Florida, 2004 Mowry Road; Room 2236-5, PO Box 100177, Gainesville, FL 32608 USA; 2 Department of Pediatrics, School of Medicine, University of Colorado, 12474 E. 19th Avenue, Building 402, Room 219 Main Stop F426, Aurora, CO 80045 USA; 3 Department of Health Outcomes and Biomedical Informatics, College of Medicine, University of Florida, 2004 Mowry Road; Room 2245, PO Box 100177, Gainesville, FL 32608 USA; 4 Department of Health Outcomes and Biomedical Informatics, College of Medicine, University of Florida, 2004 Mowry Road; Room 2244, PO Box 100177, Gainesville, FL 32608 USA

**Keywords:** Replicability, Generalizability, Subgroup, Electronic health record

## Abstract

Although superficially similar to data from clinical research, data extracted from electronic health records may require fundamentally different approaches for model building and analysis. Because electronic health record data is designed for clinical, rather than scientific use, researchers must first provide clear definitions of outcome and predictor variables. Yet an iterative process of defining outcomes and predictors, assessing association, and then repeating the process may increase Type I error rates, and thus decrease the chance of replicability, defined by the National Academy of Sciences as the chance of “obtaining consistent results across studies aimed at answering the same scientific question, each of which has obtained its own data.”[1] In addition, failure to account for subgroups may mask heterogeneous associations between predictor and outcome by subgroups, and decrease the generalizability of the findings. To increase chances of replicability and generalizability, we recommend using a stratified split sample approach for studies using electronic health records. A split sample approach divides the data randomly into an exploratory set for iterative variable definition, iterative analyses of association, and consideration of subgroups. The confirmatory set is used only to replicate results found in the first set. The addition of the word ‘stratified’ indicates that rare subgroups are oversampled randomly by including them in the exploratory sample at higher rates than appear in the population. The stratified sampling provides a sufficient sample size for assessing heterogeneity of association by testing for effect modification by group membership. An electronic health record study of the associations between socio-demographic factors and uptake of hepatic cancer screening, and potential heterogeneity of association in subgroups defined by gender, self-identified race and ethnicity, census-tract level poverty and insurance type illustrates the recommended approach.

## Introduction

Health scientists often seek to build statistical models to assess associations between predictors and outcomes using data from electronic health records. Achieving scientific standards of evidence demand satisfactory answers to two questions, one about replicability, the other about generalizability. Replicability, as defined by the National Academy of Sciences is the chance of “obtaining consistent results across studies aimed at answering the same scientific question, each of which has obtained its own data.”[1] Generalizability indicates that the results apply to multiple populations. In the context of electronic health record data, replicability implies that other scientists will reach similar conclusions if they collect similar data from a similar population and test similar hypotheses. In turn, generalizability implies similar conclusions will hold for electronic health records from different groups of people, locations and times.

Preliminary data analysis is often required for predictor or outcome variable definitions in electronic health records. This preliminary data analysis may increase decision errors. If multiple re-analyses are done, but only one is reported, the decision error rate is likely higher than claimed [2]. In a randomized controlled clinical trial or in an observational study, scientists can specify hypotheses and variable definitions in the design phase, and analyze data according to a carefully defined plan. In contrast, researchers using electronic health records must use sets of data that are constantly expanding. Instead of pre-planned, carefully defined variables, researchers must study variables which have been collected for clinical, rather than research use. The nature of the data can lead to multiple preliminary analyses, including re-defining variables of interest based on complex medical coding schemes [3], recoding informatively missing data [4, 5], and choosing different variables for modeling based on exploratory results.

Analysis of electronic health records is a special case of observational health research. Observational research often includes exploratory analyses to select the final model. These include, but are not limited to, creating new variables, checking data distributions, tabulating and modeling missingness, checking model assumptions, and applying model selection strategies to find preferred predictors. In addition, exploratory analysis also includes examining sensitivity of conclusions to extreme values or a change in cohort definition.

Exploratory, as distinct from planned, subgroup analyses have been criticized for lacking replicability [6]. All exploratory analyses increase the risk of a Type I error, which reduces replicability [7]. The process of exploratory analysis introduces optimistic bias in the estimates of effect size. The bias arises in two ways. First, exploratory analysis inflates the Type I error rate, which increases the chances of including useless variables in a model. Second, adding variables can only increase a measure of model strength, such as R^2^ or area under the curve. A preference for larger effects increases the chances of publication, which leads to publication bias. Establishing replicability in the presence of exploratory data analysis requires specific strategies.

Because electronic health records are often drawn from multiple clinical settings, they often include data from more diverse groups of people than traditional clinical studies. The inclusion of diverse groups of people can allow greater generalizability, but require careful thought about possible heterogeneity of associations, based on group membership.

Electronic health record data allow investigators to assess subgroup effects in heterogeneous samples. One way to assess the heterogeneity of the association between the exposure and the outcome across subgroups is to test the interaction between the exposure and the subgroup indicator [8]. Being able to fit the interaction model and compare subgroups requires adequate sample sizes in the smallest subgroups.

Luckily, studies of electronic health records allow collecting data from different populations, in order to explicitly test for generalizability. Collecting data from different populations has two advantages. First, the total possible variability of the target population is more likely observed in the sample. Second, the large sample size means that the sample size of subgroups of interest are large enough to allow testing subgroup differences.

Our recommendation of a split sample approach arises from our experience that research using electronic health records can lead to substantial exploratory analyses, including many model selection steps. Picard and Cook [2] provided a rationale for using a split sample analysis when extensive model selection is conducted. The unique strength of the split-sample approach lies in the ability to account for any sort of exploratory analysis.

**Fig. 1:**
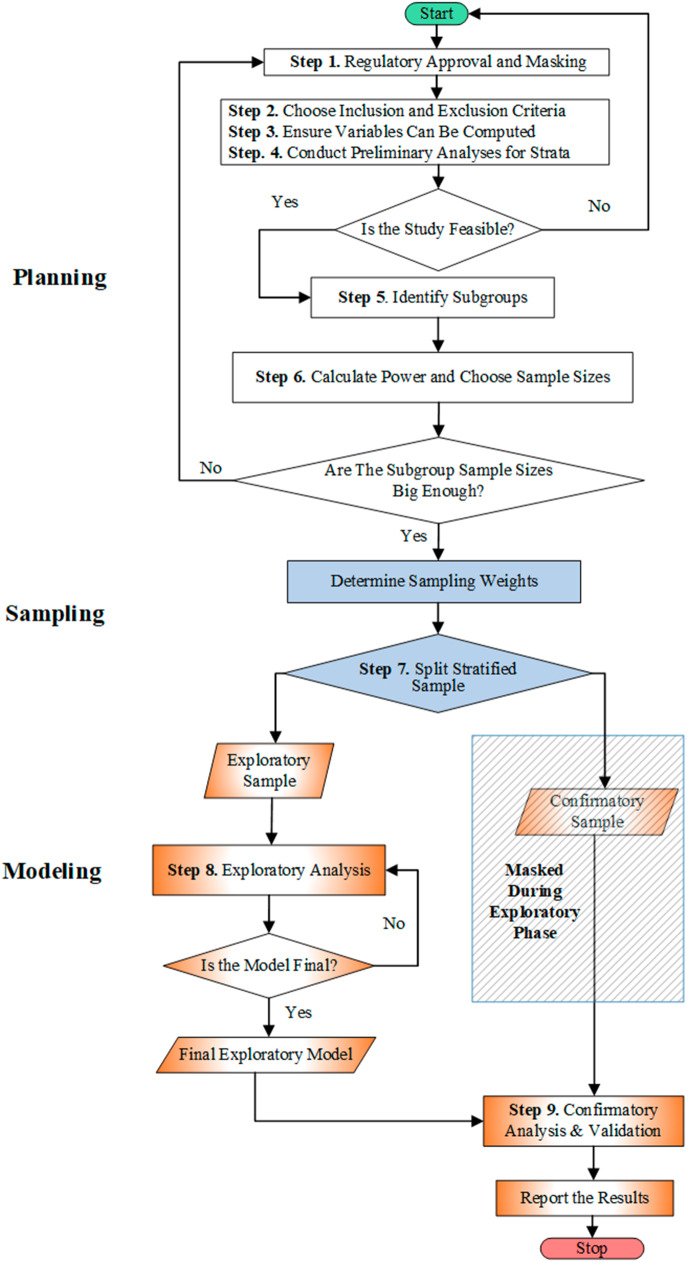
Flow chart for the process of building a model with a stratified split-sample approach

In this article, we propose a design strategy for the analysis of electronic health record data called the stratified split-sample approach, reviewed by Muller and Fetterman [8] (Sect. 11.7.3). Figure 1 contains a flow chart for the process of building a model with a stratified split-sample approach Briefly, a stratified split sample approach involves two strategic design approaches. The data is randomly split into an exploratory set for preliminary analyses, and a confirmatory set to assess replicability. Small groups are sampled at higher probability than they occur in the population. The oversampling in subgroups can permit definitive exploration of heterogeneity in subgroups, helping ensure generalizability.

## Steps for Conducting Data Analysis using Electronic Health Record Data

### Step 1. Obtain regulatory approval and decide on masking

The ethics of science require not only access to data but also appropriate permissions, such as from an institutional review board, to analyze and publish results. Privacy considerations may limit the level of detail that may be analyzed or published, such as the identity of providers and health systems.

Masking (also called “blinding”) of investigators, participants or data analysts is a key approach in clinical trials and other scientific settings to prevent biases [9].Investigators and data analysts conducting an exploratory analysis are typically unmasked in order to capitalize on their scientific knowledge.

### Step 2. Determine exclusion and inclusion criteria

Because analysis of electronic health records uses existing data, changing the inclusion and exclusion criteria is fairly low cost. It requires only a change in the code used to extract records from a database. However, if the criteria are changed after sample splitting and exploratory data analysis has begun, and the investigators redo the file extraction, the process must be reported. Although there may be no apparent effect on the conclusions, the fundamental need for transparency requires documenting the change. A similar requirement holds for changing inclusion and exclusion criteria in a clinical trial.

In some cases, it is too costly to include all available participants. When human, rather than computer-driven medical record review is used, each additional participant included incurs costs. This means that the costs of a medical record review may limit the size of a sample that meets the gold standard of data quality [10].

### Step 3. Ensure variables of interest exist, or can be computed

While working with electronic health records, investigators must be certain whether the set of data includes all the variables needed. Investigators can get the information from a data dictionary, a common data model, or meta-data. However, the presence of a coded variable in the data dictionary does not guarantee the data elements are in the file.

In some cases, creating a variable which accurately reflects a diagnosis or clinical treatment may require a careful transformation of two or more diagnosis and procedure codes. The approach is often used in health services research and biomedical informatics, and may be referred to as creating a “computable phenotype” [11].

### Step 4. Perform preliminary analyses to obtain information for the stratified split sample approach

Analyses prior to random division of the sample into an exploratory set and a confirmatory set should be limited to (1) cross-tabulations for planning the stratified sampling, and (2) cross-tabulations of missing value patterns of the outcomes and predictors to ensure study feasibility. Any cross-tabulation of predictor and outcome values should only be done in the confirmatory set, after the final model has been chosen. Computing cross-tabulations of predictor and outcome values on the full dataset would contaminate the validity of the split-sample process.

### Step 5: identify the subgroups of interest

Subgroups in a population are typically defined by socio-demographic distinctions which may be associated with both the outcome of interest and the predictor. Examples include division into groups identified by sex-assigned-at birth, by self-identified race or ethnicity, or by urban or rural residence. NIH guidelines [12] require both design and analysis of research to address the distribution of participants by self-identified gender and self-identified race or ethnicity.

Simpson’s paradox shows that ignoring important subgroups when studying associations between predictor and outcomes may create bias. Thus, identifying subgroups which may affect estimates of association is important. Oversampling subgroups, at higher rates than they may appear in the population, provides sufficient sample size for estimation and hypothesis testing.

For analysis, we suggest including data from all subgroups in a common model, and assessing interaction between subgroup indictor variables and other predictors. The analytic approach allows estimation of the magnitude and direction of associations between predictors and outcome in each subgroup, and averaged across subgroups. In addition, the analytic approach allows estimation of the magnitude and direction of differences between subgroups of strengths of associations between predictors and outcome. Finally, the analytic approach allows estimation of interactive effects between subgroup variables, and other predictors. When using stratified sampling to oversample subgroups, we note that analysis weights must be chosen carefully to provide tests and estimates that correctly reflect the scientific purposes of the study.

An alternative analytic approach for subgroup analysis is to divide the population into subgroups, and then fit a separate model to assess associations between predictors and outcomes in each subgroup [13]. The common model approach described in the previous paragraph provides greater statistical power than fitting a separate model for each subgroup [14]. Furthermore, separate models do not provide a test of interaction, which can be a more important question. For example, does the association differ for men and women?

### Step 6. Calculate power and select sample size

Sample size selection depends on the context of the study. With observational research, investigators typically include all of the available data to maximize statistical power. However, pre-specified inclusion and exclusion criteria may yield a smaller analytic dataset, even if the repository is large. For example, although a data repository may include millions of people, there may be only dozens of people having small cell lung cancer. Therefore, an estimate of statistical power is needed for the hypothesis of interest, particularly for tests of interactions involving subgroups with small sizes. With a split-sample approach, ideally the sample size chosen should be selected to ensure sufficient power to test the pre-specified hypothesis in both the exploratory and the confirmatory samples. How to estimate power is discussed in multiple other publications (see, e.g., [15]), and is beyond the purview of the current manuscript.

### Step 7. Conduct Stratified Sampling

After checking the sample size, the whole sample is split into two parts, an exploratory sample and a confirmatory sample. To prevent unauthorized exploratory analysis in the confirmatory set, a usual approach is to mask investigators and analysts so that they cannot see the confirmatory sample before all exploratory analysis is complete. All the exploratory analyses are conducted using only the exploratory sample, leaving the confirmatory sample untouched. We recommend using a stratified sampling approach to ensure the proposed subgroup effects and interactions can be estimated and tested in both the exploratory and the confirmatory samples. The variables for stratification must be chosen carefully since they cannot be changed once the stratified sampling process is completed.

There are two important factors to consider in choosing a stratification scheme. First, the size of the total sample limits choices. The smallest of the exploratory and confirmatory sample sizes must be large enough to allow estimating and testing the effects of interest. Second, the smallest cell in the subgroups of interest must be large enough in both samples. Ideally, both samples have enough statistical power to detect interactions involving variables defining subgroups.

### Step 8. Conduct exploratory analysis using only the exploratory sample

All statistical analyses and data manipulations conducted using the exploratory sample are exploratory analyses. In some cases, scientists may feel the need to conduct a series of exploratory analyses in an iterative fashion. For example, if including a particular variable creates an issue with model convergence, the variable could be dropped from the model. Alternatively, iteratively redefining a variable and checking model fit diagnostics may lead to a well-behaved model. We will not discuss in detail how to conduct an exploratory analysis, since there is extensive literature on good practice of conducting analysis on observational healthcare data by other authors [8, 16]. The approach depends on the specific medical fields [17, 18].

For model building, we recommend a five-step strategy described by Cheng et al.[19] for mixed models, which generalizes to other types of regression models. The five steps are: (1) specify the maximum model including all the main effects and interaction terms of interest, (2) specify a criterion of the goodness of fit of a model, such as model R^2^ or area under the curve, (3) specify a predictor selection strategy, such as backward elimination (which we recommend), (4) conduct the analysis, and (5) evaluate the replicability of the model chosen.

### Step 9. Conduct Model Validation and Confirmatory Analysis

There are two basic approaches to validate the exploratory results in the confirmatory sample. One common approach conducts hypothesis tests, while another evaluates prediction accuracy. For example, Muller and Fetterman 2002 [8] gave recommendations for a univariate model with Gaussian errors, and suggested computing shrinkage, the difference in R^2^ for the model fit to the exploratory data, and the same model (with the same parameters), evaluated in the confirmatory set. A statistically independent confirmatory sample allows accurately estimating shrinkage.

No matter what approach is taken, it is important to report the planned measures of scientific importance such as, for example, areas under the curve, odds ratios or R^2^. It is important to report the results, whether the exploratory results replicate or not.

## An example analysis with stratified split-sampling

We illustrate our recommended strategy for building models with electronic health records by considering a study to examine disparities in the receipt of recommended screening for hepatocellular carcinoma, the ninth leading cause of cancer deaths in the United States in 2016. Subgroups were based on gender, self-identified race and ethnicity, place of residence, behavioral and physical health co-morbidities, and access to a gastrointestinal specialist. The project used the OneFlorida Data Trust [20], a repository of healthcare information for roughly 19 million Floridians. Patient data were derived from the electronic health records of 11 hospital systems and Florida’s Medicaid system.

The goal was to evaluate factors associated with hepatocellular carcinoma screening patterns among individuals with cirrhosis. The study used a cross-sectional design to examine disparities related to subgroup membership in the receipt of recommended hepatocellular carcinoma screening. A total of 10,775 adults with cirrhosis were identified in the OneFlorida Data Trust [20] from 2014 to 2016.

The analysis pooled all data from all health care settings. Next, the analysis split the total sample into two parts, an exploratory sample and a confirmatory sample. The analysis used non-uniform stratified sampling to ensure adequate sample sizes of all subgroups of interest in both samples. Individuals were stratified by self-identified race and ethnicity, as well as population density ≥ 100 per square mile. Some self-identified race and ethnicity categories are uncommon in low population density areas. How we adjusted the sample sizes is specified later in this section. The identities of settings were masked for confidentiality reasons. We developed a regression model adjusting for the setting, which greatly improved the generalizability of the model.

Candidate predictor variables for the exploratory analyses included gender, self-identified race and ethnicity, physical or behavior comorbidities, access to a gastrointestinal specialist, insurance type and rurality. Candidate predictor interactions included rurality with each of gender, self-identified race and ethnicity, census-tract level poverty and insurance type. The outcome was a binary indicator of whether a patient received any screening.

We began by tabulating the number of screening events to create the outcome variable in the total sample. We were scrupulous to *not* cross-tabulate the outcome values with any predictor values, although we had cross-tabulated missing patterns to ensure an adequate sample size. Finding a clinically meaningful and computable specification of cirrhosis required a dozen iterations. The process led us to refine the rule for cohort inclusion criteria.

The next step was the cross-tabulation of subgroup memberships, i.e. rurality, self-identified race and ethnicity and gender. In rural areas, Hispanics were only 0.5% (n = 56), non-Hispanic blacks 0.7% (n = 80) and non-Hispanic others 1% (n = 103) of the total cohort (n = 10,775), which raised concern about the ability to estimate the self-identified race and ethnicity by rurality interaction. Being able to compute an unbiased estimate of a subgroup property, such as a mean or proportion, depends only on the subgroup size. However, finding unbiased estimates of regression coefficients for subgroup main effects and interactions partially depends on the relative frequencies (percentages) of the subgroups. Subgroup percentages that are too small can induce problems with collinearity (Muller and Fetterman, Chap. 8) [8]. Therefore, in the exploratory sample, we used unequal sampling rates. We allocated 90% of rural residents and 10% of urban residents to the exploratory sample. Doing so guaranteed at least 50 persons in each self-identified subgroup, cross classified by self-identified race, ethnicity and rurality. The exploratory sample contained 2.8% self-identified Hispanics, 4.0% self-identified non-Hispanic blacks and 5.2% self-identified non-Hispanic others in rural areas.

We conducted many analyses on the exploratory sample, including tabulating descriptive statistics and refining the specification of the outcome variable. We explored three types of rurality measures: population density, the Urban-Rural Classification Scheme from the National Center for Health Statistics, and Rural-Urban Continuum Codes from U.S. Department of Agriculture, by including the measure in the model one at a time. After selecting population density to quantify rurality, we started with the maximum model and conducted backward model selection, as recommended by Kleinbaum et al. [21]. We dropped the non-significant interactions and covariates to yield a final model.

Using the model coefficients estimated in the exploratory analysis, we computed predicted values for each individual for the confirmatory sample. We used the predicted values to conduct a receiver operating characteristic analysis to confirm the robustness of the exploratory model. The area under the curve did not shrink appreciably from the value in the exploratory analysis. Furthermore, the confidence interval around the confirmatory area under the curve was narrow.

Using a split sample analysis requires including details of the process in the methodology and results sections of a research report. Figure 1 provides a template for an exhibit in a manuscript. Investigators could use the figure as a checklist for a methods section which described the approach for splitting the sample, blinding, oversampling, and so forth. The associated text provides a concrete and ordered list of steps to describe. The example includes brief illustrations of the steps.

## Discussion

Our principal findings address the path to achieving replicable research when using electronic health records, which require exploratory analysis to specify outcome and predictor variables. The need for exploratory analysis presents a challenge in achieving replicable conclusions. A non-iterative split-sample design protects the replicability of data analyses that include exploratory steps. Stratified sampling of electronic health records protects generalizability by allowing heterogeneity between subgroups to be tested appropriately with the best statistical power available with the data. Non-iterative split-sample designs are needed to protect replicability and generalizability in the presence of extensive exploratory analyses.

A data-driven choice is a decision or a definition based on a particular set of data. An example might be defining obesity as a BMI above a sample, rather than a population, quantile. When the data drive the decision, an analyst evaluating a similar but distinct set of data may have their data make a different decision, and end up with a different answer. Analysis of electronic health records can lead to changing variable and model specifications, and adding predictors to accommodate unexpected scenarios.

The presentation differs from previous discussions by emphasizing the primacy of replicability as a goal requiring special attention. Our recommendations differ from those in the TRIPOD statement [22], which does not consider studies needing extensive exploratory analyses, as is typical with research on electronic health records.

An example analysis of electronic health records illustrates the approach. Given that the exploratory process can make the distribution of a statistic from the exploratory sample uncertain, we did not conduct a statistical test to compare the model fits of the exploratory and the confirmatory samples. Many authors recommend doing so [23].

We hope scientists conducting research using electronic health records will adopt our recommendations. In addition, we hope readers, reviewers, editors and policymakers will judge research using electronic health records in the light of our recommendations.

## Data Availability

The datasets used and/or analyzed during the current study available from the corresponding author on reasonable request.
